# Neonatal Imitation in Rhesus Macaques

**DOI:** 10.1371/journal.pbio.0040302

**Published:** 2006-09-05

**Authors:** Pier F Ferrari, Elisabetta Visalberghi, Annika Paukner, Leonardo Fogassi, Angela Ruggiero, Stephen J Suomi

**Affiliations:** 1 Dipartimento di Biologia Evolutiva e Funzionale, Università di Parma, Parma, Italy; 2 Dipartimento di Neuroscienze, Università di Parma, Parma, Italy; 3 Istituto di Scienze e Tecnologie della Cognizione, National Research Council, Rome, Italy; 4 Laboratory of Comparative Ethology, The National Institute of Child Health and Human Development, Bethesda, Maryland, United States of America; 5 Dipartimento di Psicologia, Università di Parma, Parma, Italy; Emory University, United States of America

## Abstract

The emergence of social behaviors early in life is likely crucial for the development of mother–infant relationships. Some of these behaviors, such as the capacity of neonates to imitate adult facial movements, were previously thought to be limited to humans and perhaps the ape lineage. Here we report the behavioral responses of infant rhesus macaques *(Macaca mulatta)* to the following human facial and hand gestures: lip smacking, tongue protrusion, mouth opening, hand opening, and opening and closing of eyes (control condition). In the third day of life, infant macaques imitate lip smacking and tongue protrusion. On the first day of life, the model's mouth openings elicited a similar matched behavior (lip smacking) in the infants. These imitative responses are present at an early stage of development, but they are apparently confined to a narrow temporal window. Because lip smacking is a core gesture in face-to-face interactions in macaques, neonatal imitation may serve to tune infants' affiliative responses to the social world. Our findings provide a quantitative description of neonatal imitation in a nonhuman primate species and suggest that these imitative capacities, contrary to what was previously thought, are not unique to the ape and human lineage. We suggest that their evolutionary origins may be traced to affiliative gestures with communicative functions.

## Abstract 2

This manuscript provides the first quantitative description of neonatal imitation in a nonhuman primate, indicating imitative capacities are not unique to the ape and human lineage, contrary to what was previously thought.

## Introduction

Matching one's own behavior with that of others allows individuals to detect contingencies in the social world. This process could allow an individual to synchronize its activity with those of its group members, to copy the behavior of other individuals, and to learn the context in which an activity should be performed [1,2]. Tracking signs of this phenomenon early in life is important to understand its development and the biological features eliciting it.

To date, studies of early signs of this matching capacity have been largely limited to human infants. Almost 30 years ago, Meltzoff and Moore [3] reported that 2- to 3-wk-old infants responded with corresponding matching behaviors to specific human facial gestures, such as mouth opening (MO), tongue protrusion (TP), lip protrusion, and hand opening (HO). Other studies confirmed this early investigation, although there is still considerable debate about which gestures are actually imitated [4–9]. To avoid the possible interferences of early learning experiences with innate imitation processes, Meltzoff and Moore conducted further investigations immediately after birth and demonstrated that newborns also can imitate adult facial gestures [4,5]. They argued that the specificity of the imitative response indicates a capacity to accurately match the body parts involved. Because newborns cannot see their own face but can only perceive it through proprioception, the matching of their own acts to those observed should require a supramodal representation of the observed gesture, called active intermodal matching [3–5,10].

We know very little about the evolutionary origin of this capacity. Recently, Matsuzawa and colleagues studied neonatal imitation in two infant chimpanzees *(Pan troglodytes)* that had been reared from birth by their biological mothers. The results were similar to those obtained with human infants [11]. Both infant chimpanzees imitated human facial gestures such as TP and MO within the first week of life, confirming previous observations carried out in a single subject by Myowa [12]. The chimpanzees' ability to imitate human facial gestures disappeared after 2 mo in both studies, similar to what has been reported for human infants [13,14]. Independently, another study with five neonate chimpanzees aged less than 3 d confirmed imitation of MO and TP [15].

Although neonatal imitation in chimpanzees, and especially in other ape species, requires further investigation, it seems that this phenomenon has features similar to human neonatal imitation, both in terms of timing and type of imitated gesture. This observation is congruent to the finding that humans and apes appear better endowed for imitation than are other primate species [15–18]. Studying neonatal imitation in a more evolutionary distant primate species in which the imitative processes are usually not present [19] might provide insights about how, when, and why this phenomenon evolved. This empirical strategy may have the dual advantage of marking possible cognitive boundaries between our species and other primates and, at the same time, delineating possible common elements shared by monkeys, humans, and chimpanzees.

To pursue this goal, we investigated the presence of neonatal imitation in rhesus macaques, an Old World monkey species that diverged from the human lineage about 25 million y ago [20]. This study represents the first detailed analysis, to our knowledge, of neonatal imitation conducted in a primate species outside the great ape clade. We provide evidence that infant macaques imitate mouth gestures (TP and LPS) performed by a human experimenter and that the temporal window in which this capacity is present is likely limited to the first days after birth. The results indicate that the capacity of neonates to imitate facial gestures may not be an evolutionary acquisition of apes and humans alone.

## Results

**Figure 1:**
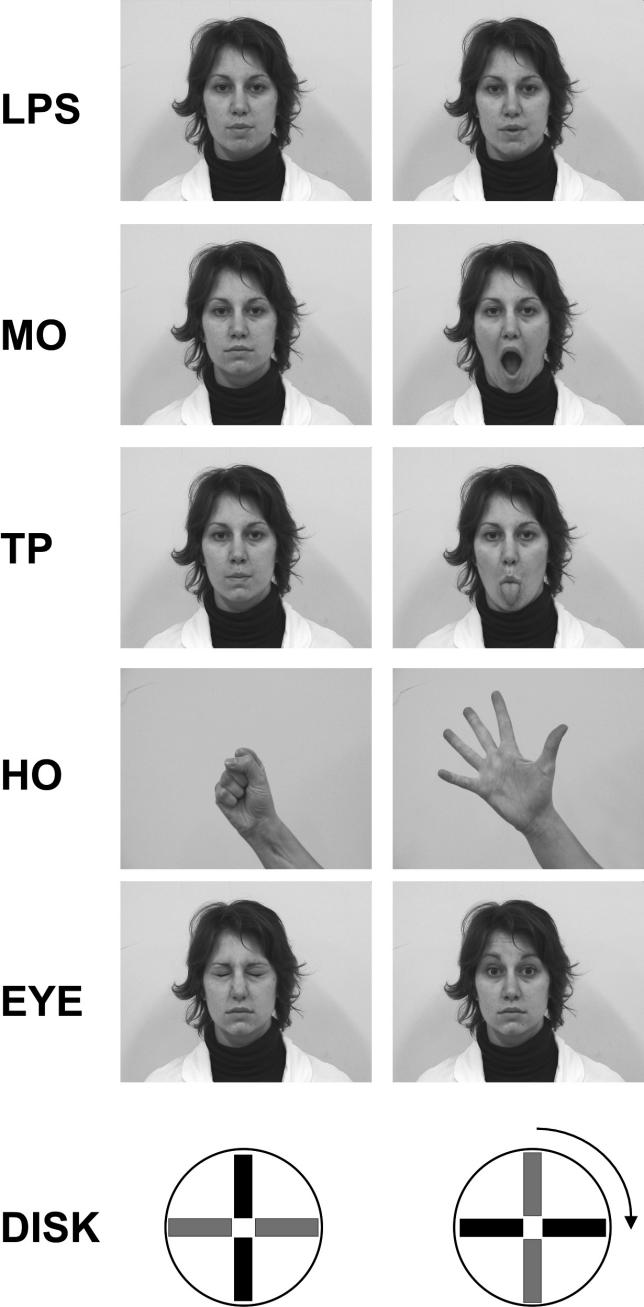
Experimental Conditions Figures on the left represent stimuli during resting conditions and baseline. Figures on the right depict the stimuli when fully expressed. In the DISK condition, the disk was repeatedly rotated 90° clockwise and counterclockwise.

We tested 21 infant rhesus macaques at ages of 1, 3, 7, and 14 d. Infants were tested once a day in six different conditions (Figure 1). Each condition consisted of two time periods: baseline (40-s duration) and stimulus (20 s of stimulus presentation followed by 20 s of passive face). During baseline, the experimenter faced the infant with a passive/neutral facial expression. During stimulus presentation, one of the following gestures was performed by the experimenter: TP, MO, LPS, HO, or eyes opening (EYE, biological control condition). An additional control condition involved a nonbiological stimulus (DISK, a disk rotating clockwise and counterclockwise) to assess infants' attention toward biological versus nonbiological stimuli.

We videotaped the infants' behavior in each condition and analyzed whether their behavior matched the stimulus. We compared the frequency of matched behaviors in the baseline and stimulus periods and the frequency of the matched behaviors in each specific condition with the corresponding behavior in the biological control condition (EYE).

### Infants' Attention (LOOK) to the Stimulus

**Figure 2:**
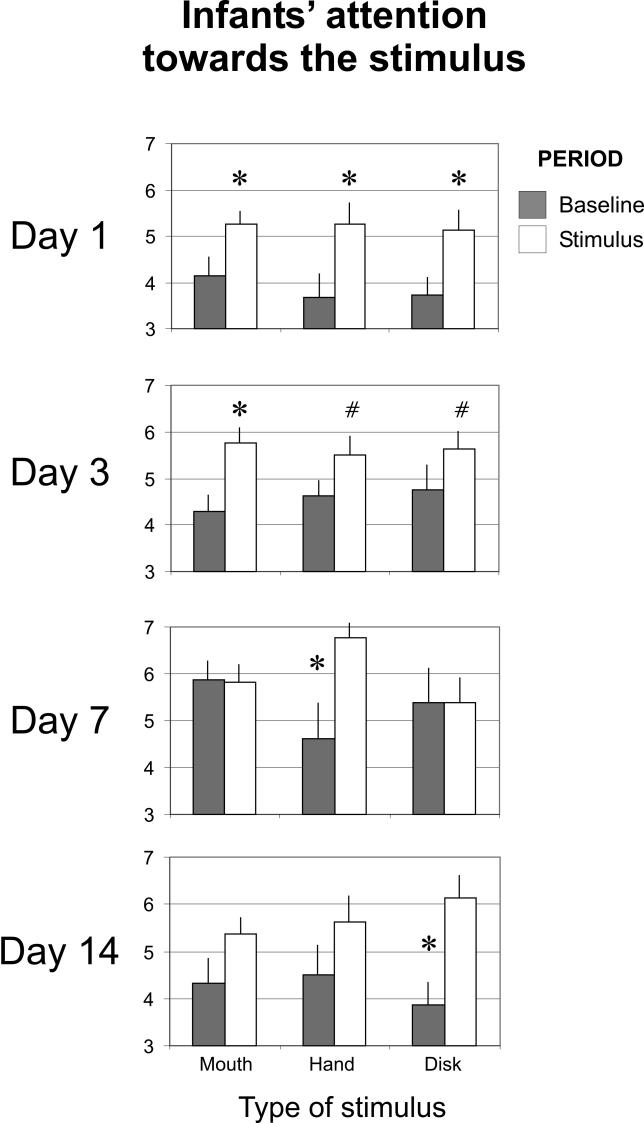
Frequencies of Looks That the Infants Oriented at the Stimulus during the Baseline and the Stimulus Period Asterisks (*) indicate a significant increase in number of looks (stimulus versus baseline) for a specific stimulus (at least *p* < 0.05). Pound symbols (#) indicate that this effect is close to statistical significance (*p* < 0.07). Because data concerning looks at models' LPS, MO, and TP were very similar, they were averaged and pooled. Frequencies are ± standard error of the mean.

During both baseline and stimulus periods, the amount of attention paid to the experimenter face/stimulus during the presentation of biological (mouth, tongue, eyes, and hand) and nonbiological stimuli (disk) did not differ among conditions. In general, the infants looked more at the stimuli during the stimulus period than at the baseline (Figure 2), although this effect was less robust on days 7 and 14. More specifically, this effect was statistically significant or close to significance on day 1 (mouth: *z* = 2.73, *p* < 0.01; hand: *z* = 2.33, *p* < 0.02; disk: *z* = 2.17, *p* < 0.05; eyes: not significant) and day 3 (mouth: *z* = 2.76, *p* < 0.01; hand: *z* = 1.82, *p* < 0.07; disk: *z* = 1.86, *p* < 0.07; eyes: *z* = 2.10, *p* < 0.05). On day 7, this effect was not present except in the hand condition (*z* = 2.17, *p* < 0.05). On day 14, the attention toward the stimulus was greater during the stimulus period than in the baseline in the DISK (*z* = 2.88, *p* < 0.005) and the EYE conditions (*z* = 2.55, *p* < 0.01).

### Infants' Response to the Biological Stimuli

**Figure 3:**
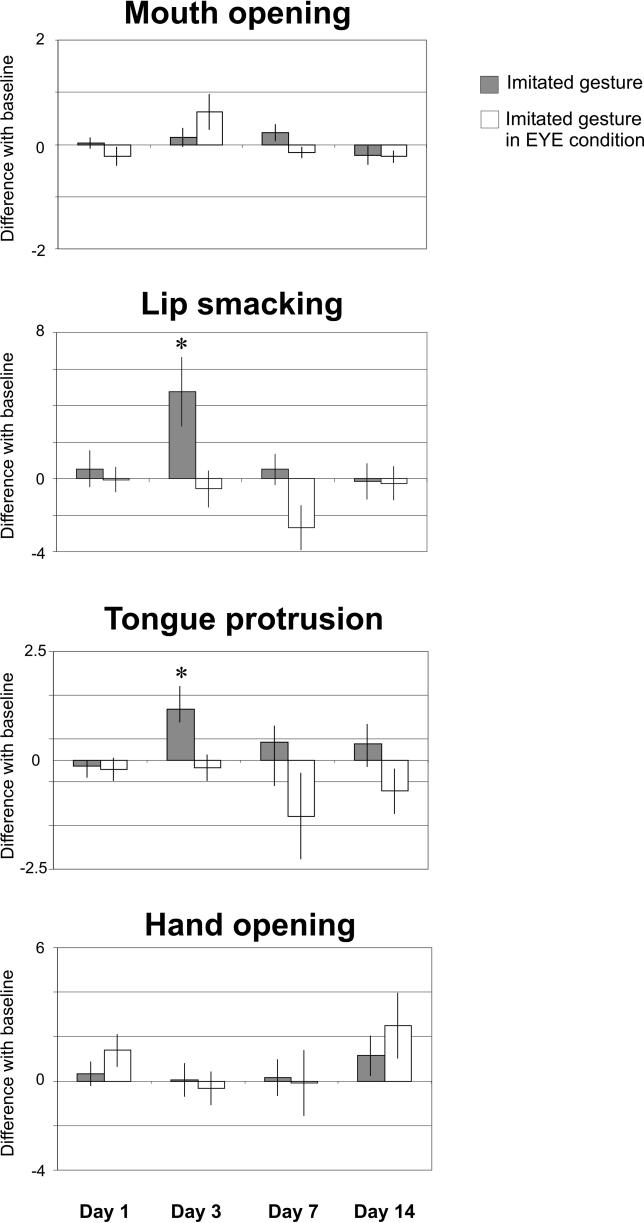
Averaged Scores of the Imitated Behaviors and the Same Behaviors Scored in the Control Condition (EYE) Averaged scores are calculated as the difference between the frequency of the imitated behavior in the stimulus period and the baseline. The scores the infants obtained are reported in relation to age and to the different experimental conditions (MO, LPS, TP, and HO). Scores are ± standard error of the mean.

Figure 3 illustrates the infant macaques' responses to human facial and hand gestures at different ages. We report only results that obtained statistical significance.

On day 1, the frequency of MOs made by infant macaques was very low or absent (mean number of MOs during stimulus period in the different conditions were 0.33 in MO, 0.41 in TP, 0.47 in LPS, 0.13 in HO, and 0.07 in EYE). In contrast, high rhythmic mouth openings/closures (defined as LPS) were frequent. On day 1, in the MO condition, the frequency of MOs in the stimulus period was not different from baseline, although the frequency of LPS (in the MO condition) was significantly higher (*z* = 2.36, *p* < 0.02). More specifically, for eight out of 15 individuals, LPS increased in the stimulus period compared with baseline, whereas for one infant, it decreased and for six infants, no change occurred. The increase in LPS between baseline and stimulus periods tended to be greater in the MO condition than in the EYE condition (*z* = 1.77, *p* < 0.075), and it was significantly higher than in the TP (*z* = 2.35, *p* < 0.02) and HO (*z* = 2.20, *p* < 0.03) conditions. In the MO condition, the frequencies of HOs, MOs, and TPs did not increase in the stimulus period compared with the baseline. No significant changes were detected in any of the other conditions.

**Figure 4:**
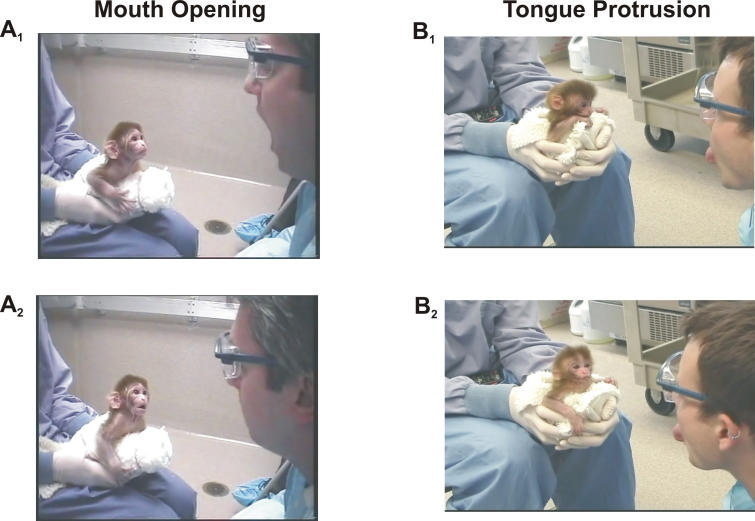
Two Examples of a Monkey's Response to the Stimuli Mouth Opening and Tongue Protrusion MO is shown on the left; TP on the right. Figures were taken from Videos S1 and S2. Frame A1 was taken 21.12 s after frame A, whereas frame B2 was taken 13.38 s after frame B.

On day 3, the frequency of TPs in the TP condition and of LPS in the LPS condition were significantly higher during the stimulus period than during baseline (*z* = 2.19, *p* < 0.03 and z = 2.23, *p* < 0.03, respectively). The increases of LPS in the LPS condition and of TP in the TP condition were significantly higher than their respective increases in the EYE condition (*z* = 2.04, *p* < 0.05 and *z* = 2.26, *p* < 0.03, respectively). Figure 4 (and Videos S1 and S2) provides examples of two macaques responding to the experimenter's MO (left) and TP (right). In the LPS condition, ten out of 16 individuals increased the frequency of LPS in the stimulus period (in three individuals there was no change and in three, it decreased). In the TP condition, eight out of 16 individuals increased TPs in the stimulus period (in six infants there was no change and in two others, it decreased). Only five subjects increased both LPS in the LPS condition and TP in the TP condition.

**Figure 5:**
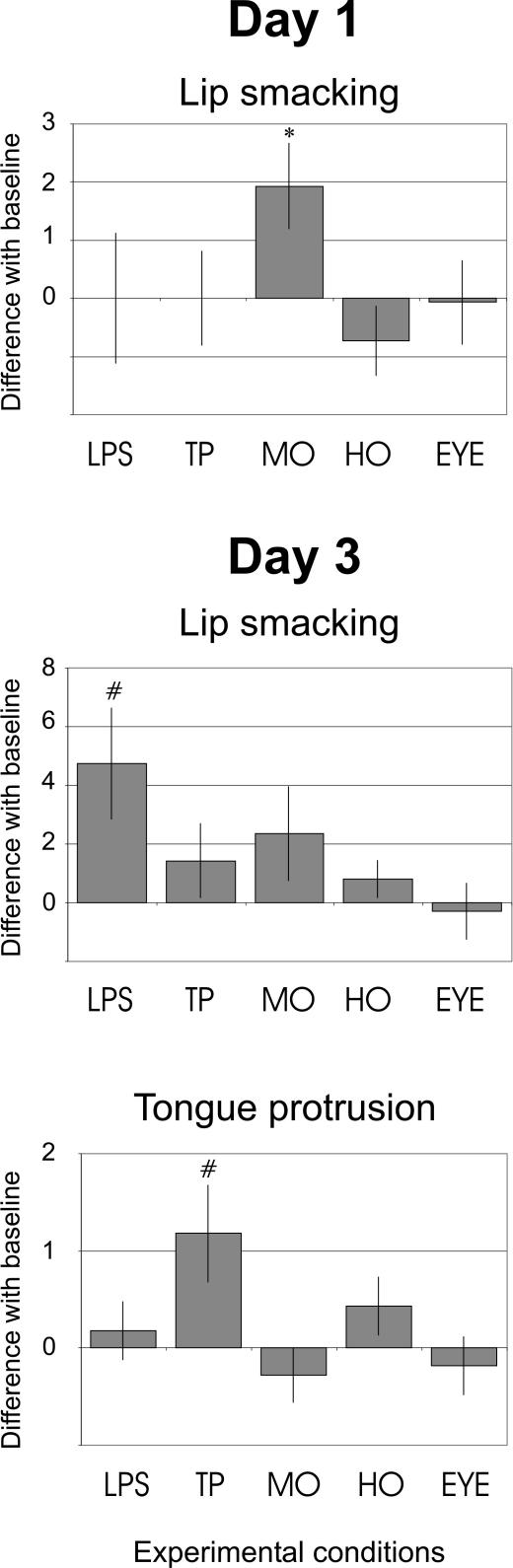
Averaged Scores of Lip Smacking on Days 1 and 3 and of Tongue Protrusion on Day 3 and of the Same Behaviors Scored in the Other Experimental Conditions Averaged scores are calculated as the difference between the frequency of the imitated behavior in the stimulus period and the baseline. The asterisk (*) indicates a significant difference of LPS score in the MO compared with TP and HO conditions. Pound symbols (#) indicate a significant difference of the behavioral score recorded in that condition compared with all the other conditions. Scores are ± standard error of the mean.

Figure 5 illustrates the frequencies of LPS on day 1 and of LPS and TPs on day 3 in all experimental conditions. The frequencies of LPS in the LPS condition and of TP in the TP condition were higher than the frequencies of those same behaviors in all the other conditions (LPS frequency in LPS versus MO condition: *z* = 1.95, *p* < 0.05; versus TP condition: *z* = 2.19, *p* < 0.03; versus HO condition: *z* = 1.98, *p* < 0.05; versus EYE condition: *z* = 2.31, *p* < 0.02. TP frequency in TP versus MO condition: *z* = 2.36, *p* < 0.03; versus LPS condition: *z* = 2.54, *p* < 0.01; versus HO condition: *z* = 1.95, *p* < 0.05; versus EYE condition: *z* = 2.65, *p* < 0.01).

In the LPS condition, the frequencies of HOs, MOs, and TPs did not increase in the stimulus period compared to the baseline. In the TP condition, the frequencies of HOs, MOs, and LPSs did not increase in the stimulus period compared to the baseline.

On day 7, there was a tendency to perform more LPS in the LPS condition than in the EYE condition (*z* = 1.77, *p* < 0.08). On day 14, no differences were detected between the two periods in any condition.

## Discussion

Our findings show that 3-d-old macaques imitate LPS and TP when seeing these gestures being performed by a human experimenter. On day 1, the MO stimulus elicited a significantly higher frequency of MOs in terms of lip smacks (repeated MO) but not in terms of the exactly matched behavior (a single MO). Thus, infants matched the type of behavior in the form but not in the pattern (repetition of mouth opening/closure). This finding could be attributed to several factors that are not mutually exclusive. First, the frequency of infants' spontaneous MOs was very low or virtually absent not only on day 1 but also on any other testing day. In contrast, as shown by our data, LPS is much more frequently displayed soon after birth than is MO, and it could be considered an easier behavior to match the MO of the model. Second, the visual system of the infant is not fully developed immediately after birth, and thus the model's MO might provide a much more visible and salient stimulus than LPS because, although both share some visible features, MO (contrary to LPS) involves a wide opening of the mouth. Thus, the infant could recognize the model's MO as a form of LPS behavior and, consequently, might respond to it.

Our findings cannot be interpreted in terms of a general, nonspecific arousal response of the infant to the observation of mouth or hand gestures for the following two reasons: (i) because the increase of a specific behavior was recorded only in the matching condition; i.e., TP increased only in the TP condition; and (ii) because we did not find increased frequencies of all the behaviors, regardless of whether they matched that performed by the model, as a generic arousal model will predict.

By day 7, neonatal imitation had largely disappeared, although some individuals (four out of 12) still matched the LPS. The infants were attentive to all the stimuli; nevertheless, only a few stimuli elicited neonatal imitation and only during the very first days of life. The other stimuli (i.e., hand, eyes, and disk) elicited the infants' interest but did not produce any specific change in the infants' responses. Thus, the mouth and the tongue appeared to be the only effective stimuli among those tested in producing an imitative response in these macaque infants. The lack of neonatal imitation of hand gestures reported here is in agreement with what has been found in chimpanzees [11] and also with some human studies that clearly replicated TP and MOs effects found by Meltzoff and Moore. However, we did not consistently find the same effect for the HOs [9].

Environmental rearing conditions and the unnatural source of stimuli might account for the limited number of gestures matched and the short time course in which neonatal imitation was observed. It is possible that infants that are separated from the mother at birth lack the rich social input required to adequately respond to gestures and to maintain such responses over time. Moreover, the biological stimuli provided by the experimenter were most likely less salient for monkeys than for those routinely provided by conspecifics (i.e., the mother or group members). These factors could have reduced the effectiveness of the stimulus and, consequently, the amplitude and time course of the imitative response. In addition, because the infant was not emotionally attached to the experimenter, the possible functional meaning of neonatal imitation might have been masked or could not emerge in its complexity.

Neonatal imitation in humans shows great interindividual variation [6,9]; indeed, it does not seem to appear in some infants. Furthermore, only TP and mouth gesture imitation, including emotional facial expressions, have been consistently reported across studies [6,7,21]. This imitative phenomenon, lasting 2–3 mo [6], occurs during a period in which infant humans develop new social abilities such as spontaneous vocalizing and smiling at others [22,23]. Similarly, infant chimpanzees imitate facial gestures at 1 wk of age and apparently cease to do so at 2 mo of age [11,12]. As in humans, the types of imitated gestures were MO and TP. Although Myowa-Yamakoshi et al. [11] used only two chimpanzees, it is remarkable how similar their results were to those obtained in human infants, both in terms of timing and type of matching. However, conclusions on possible homologies between nonhuman primates and human neonatal imitation, although very plausible, should be made with caution. Such similarities need to be further investigated in chimpanzees (and other ape species as well) and macaques, because the studies available are based on a limited number of subjects. Other phenomena related to neonatal imitation that have been demonstrated in humans, such as delay response and identity of the person effect, have not yet been studied in chimpanzees or other primates.

One of the main differences between the conclusions of our study and the other primate studies is the temporal window in which neonatal imitation was observed. In contrast to humans and chimpanzees, our infant macaques showed the phenomenon for only a few days after birth. As mentioned above, some individuals still displayed imitation of LPS at day 7, but not beyond that. How can such species differences be explained? Motor and cognitive development in macaques is much more rapid in macaques than in humans and chimpanzees [24–26]. Already at 1 wk, infant macaques may leave their mother for short periods of time. Infant exploration, involving mother–infant separation, increases over time. In our experiments, we noticed that holding a 2-wk-old or older infant and capturing its attention with the stimulus became more and more difficult with increasing age. In humans and chimpanzees, neonates stay in body contact with their mother for much longer, and the mother is the only one responsible for maintaining the infant. Thus, neonatal imitation in rhesus macaques occurs with a timing that, considering the species-specific patterns of development of motor and cognitive skills, is comparable with those reported for humans and chimpanzees.

Another important aspect that emerged from our study was the marked interindividual variability in neonatal imitative abilities. Attention paid to the stimulus was not predictive of matching the gesture. Some infants consistently imitated the model's gestures, whereas others did not imitate at all at any age. Interindividual variability cannot be attributed to environmental factors, because the housing and timing of testing were identical for all subjects. Rather, it might be related to differences in temperament that predisposes the sensorimotor system to be differently sensitive and reactive to external social events. However, we cannot draw any conclusion from our data on possible relations between predispositions of the sensorimotor system and stable individual traits. Longitudinal long-term studies may help in clarifying this possible relation. Finally, infants who imitated one specific gesture were not necessarily the same individuals who imitated the other gestures. Thus, the capacity to respond to the model may not reflect a general imitative skill but rather a sensorimotor sensitivity tuned to specific facial features.

A traditional notion in primate behavior is that apes imitate, and monkeys do not [19,27]. The capacity to learn new behaviors by imitation and the repetition of simple actions or movements already in the behavioral repertoire of the animal clearly represent two different phenomena [19,28–30]. On one hand, investigations focusing on the capacity to learn new behaviors by imitation in macaques and other monkeys have shown that the primates are not capable of imitating others in the sense that they do not learn a model's action that is novel to their own behavioral repertoire via imitation [19,31]. On the other hand, recent studies seem to suggest that macaques may be capable of displaying simple forms of imitation. Kumashiro et al. [32] trained Japanese macaques to perform joint attention and then to follow the experimenter's pointing gestures. By reinforcing spontaneous repetition of some gestures, the researchers taught the monkeys to match several of the experimenter's gestures (e.g., TP, clapping hands, hand clench, and touching one's own ear). Macaques and capuchin monkeys spontaneously perform actions related to food ingestion when they see conspecifics performing those same actions [33–35], and macaques recognize when they are being imitated by a human experimenter [36]. Therefore, it appears that adult macaques have a mechanism for matching another's behavior with their own. This matching mechanism would allow them to repeat an observed behavior that is already in their behavioral repertoire.

Several hypotheses have been put forward to identify which mechanisms might underlie neonatal imitation [37]. One of them, originally proposed for human neonatal imitation, claims that an active intermodal matching mechanism is responsible [3–5]. According to this model, the perception and production of acts in human neonates can be represented within a common supramodal framework, enabling neonates to process visual and motor information cross-modally and subsequently perform the matching motor response. An alternative hypothesis, based on neurophysiological findings, proposes that infant imitation results from a “resonance” mechanism [38,39] in which the motor system of the observer (or of the listener) is activated specifically by observing (or listening to) actions. This neural mechanism has been identified in a class of visuomotor neurons, termed mirror neurons, found in the macaque premotor and parietal cortex [40–42]. These mirror neurons become active both when the monkey makes a specific action with its hand (or mouth) and when the monkey observes similar hand (or mouth) actions performed by another individual. A class of mirror neurons was recently described that responds to facial communicative actions such as LPS and TP [41]. Taking into account these neurophysiological data, our findings are in agreement with the “mirror neurons hypothesis,” according to which the observation of mouth gestures directly activates similar motor programs in the monkey premotor areas, leading them to resonate and consequently to give rise to an overt replica of the observed gestures (LPS and TP). Similarly, neonatal imitation in humans can be interpreted within this hypothetical framework. In fact, several brain imaging studies support the existence of a mirror system in humans involving frontal and parietal areas that are homologous to those in which mirror neurons have been found in monkeys [2].

Meltzoff and Moore [43], on the basis of an experiment in which 6-wk-old infants remembered and imitated a gesture performed by an adult 1 d earlier, proposed that neonatal imitation can serve to identify individuals. According to other authors, neonatal imitation may attract caretaker attention, thereby increasing opportunities for social interactions [11]. Our data may help in clarifying the functional meaning of the phenomenon, because macaques also can match one gesture with an obvious meaning, such as LPS. LPS consists of rhythmic opening and closing of the mouth that may alternate with the protrusion of the tongue [44–46]. LPS in macaques is an important facial gesture communicating affiliation, usually used to reduce distance between two individuals, and it accompanies grooming sessions [44–46]. In this perspective, TP as part of the LPS gesture plays a role in dyadic communicative exchanges [46]. In fact, infant TPs accompanied by rhythmic opening and closing of the mouth are frequently displayed by infant macaques (Video S2).

Investigations of the ontogeny of communicative gestures in macaques showed that LPS begins to develop in the first few days of life [47]. In the first weeks of life, the infant's behavioral responses toward the social world are likely to be crucial for the infant to learn and respond appropriately to social gestures displayed by other individuals. We hypothesize that infant macaques imitate those affiliative facial gestures because they are the most appropriate responses for tuning their behaviors to individuals who show affiliative behaviors toward them. Clearly, the mother plays a crucial role in these dyadic exchanges. Some literature on human neonatal imitation emphasizes the communicative aspects of this phenomenon, especially in the face-to-face interactions [48,49]. We recently observed LPS exchanges between mother and infants in the first weeks of life in rhesus macaques living in a seminatural environment (Video S3). These observations suggest that these types of interactions, involving face-to-face communication, are common not only in chimpanzees and humans but also in macaques.

## Materials and Methods

### Subjects and housing.

Subjects were 21 infant rhesus macaques *(Macaca mulatta),* 14 males and seven females. To test these macaques, we took advantage of ongoing experiments requiring infants to be separated from their mother on day 1 post-partum. They were all reared in a nursery facility according to procedures described by Ruppenthal et al. [50]. Infants were housed individually in plastic cages (51 × 38 × 43 cm), which contained a 25-cm-high inanimate “surrogate mother,” composed of a 16.5-cm-circumference polypropylene cylinder attached by a flexible metal component to an 11.5-cm-wide circular metal base. The cylinder was wrapped in an electric heating pad that was covered with fleece fabric. Loose pieces of fleece fabric also covered the floor of the cage. The incubator was maintained at a temperature of ~27 °C and at 50%–55% humidity. Lights were on from 07:00 to 21:00. Infants could see and hear, but not physically contact, other infants.

All animals were provided with a 50:50 mixture of Similac (Ross Laboratories, Columbus, Ohio, United States) and Rimilac (Bio-Serv, Frenchtown, New Jersey, United States) formulas. They were hand-fed until they were old enough to feed independently, usually by day 4. Formula was administered ad libitum until 4 mo of age. Purina High Protein Monkey Chow (#5038) (Purina, St. Louis, Missouri, United States) and water were available ad libitum when nursery-reared animals reached 1 mo of age.

All testing was conducted in accordance with regulations governing the care and use of laboratory animals and had prior approval from the Institutional Animal Care and Use Committee of the National Institute of Child Health and Human Development.

### Testing.

Subjects were tested at ages of 1, 3, 7, 14, and 30 d or, due to experimental constraints, within 1 d before or after these days. Early in the study, we found that by day 30, infants were highly mobile and difficult to hold for more than few seconds. For this reason, we abandoned the day 30 testing. Seven infants were tested at all four remaining ages, seven at only three different ages, one at two different ages (days 1 and 3), and five at one age (*n* = 3 monkeys at day 1; *n* = 1 at day 3; *n* = 1 at day 14). To summarize, we tested 15 infants at day 1, 16 at day 3, 12 at day 7, and 13 at day 14. If during testing, some animals were sleepy or too mobile, we waited for a few minutes until the infant was more awake or calm enough to be tested. However, no infants were eliminated from the analysis. Infants were tested ~30–90 min after feeding in an experimental room designed to minimize visual and auditory distractions. Once an infant was transferred to that room, a 10- to 20-min period of habituation followed to allow the infant to settle down. During testing, the experimenter was seated on a chair and held the infant while it was grasping the surrogate, or pieces of fleece fabric. This arrangement visibly calmed the infants and minimized their distress.

Three experimenters were involved in the data collection. One experimenter held the infant monkey in his/her hands, the second (the demonstrator) served as the source of stimuli, and the third videotaped the experiment and informed the demonstrator of the correct sequence of stimuli. Two video cameras (Panasonic VHS, Panasonic, Secausus, New Jersey, United States), and Sony digital, Sony, Tokyo, Japan; positioned 1.5 m lateral to the monkey) recorded the experiment. One video camera recorded both the experimenter and the infant in side view; the other recorded solely the subject's entire body from the other side (at about 120° angle from the other camera).

Each test session included six different conditions (Figure 1). Each condition consisted of two different time periods: baseline (40-s duration) and stimulus (20 s of stimulus presentation followed by 20 s of passive face). During baseline, the infant faced one of the following stimuli according to the experimental condition: (i) the demonstrator with a passive/neutral facial expression (in the conditions involving mouth gestures), (ii) the experimenter hand (in the condition involving the hand gesture), or (iii) a disk (in the DISK condition). During stimulus presentation, one of the following gestures was presented repeatedly: TP (protrusion with maximal extension and retraction of the tongue, ~seven openings/20 s), MO (opening and closing the mouth with a maximal aperture, ~seven openings/20 s), LPS (a high-frequency opening and closing of the mouth without sound production, ~100 openings/20 s), HO (opening and closing the hand, ~seven openings/20 s), EYE (opening and closing of the eyes including eyebrow lifts but without moving the lower face, ~seven openings/20 s), or DISK (a 15-cm-diameter plastic disk with a red and black cross painted on it, rotated 90° clockwise and counterclockwise). We introduced this last condition to compare the effect of a nonbiological stimulus and movement, similar in size to the human face and hand, on infant macaque behavior. On each testing day, each stimulus was presented only once. Stimuli were presented in a randomized sequence with two constraints: a mouth stimulus was never directly followed by a second mouth stimulus, and the same sequence of conditions was never repeated over two consecutive testing sessions.

### Behavioral analysis.

 Most of the tapes (80%) were digitally analyzed by two coders not blind to the experimental condition using all occurrence sampling for all behaviors listed below. Reliability between the two coders was very high (Cohen's kappa = 0.95). The analysis was not blind, to allow the coders to score the infants' behavior in relation to the beginning of each period, which was aligned to the time point in which the stimulus appeared on the screen, started to move (stimulus period), and ceased to move (post-stimulus period). However, to ensure the reliability of this procedure, 20% of sessions were coded with the human model covered on the screen so that the scorer was blind to the experimental condition. Reliability between the two coders was still high (Cohen's kappa = 0.86). The outcomes of these sessions were compared with the analysis of the same sessions in which the scorer was not blind to the experimental condition. Consistency between the blind and nonblind coding was very high (Pearson correlation: *r* = 0.879, *p* < 0.001).

The following behaviors were scored for analysis: (i) Attention to the model (LOOK). The monkey orients and looks at the stimulus (neutral face during baseline and post-stimulus, stimulus during stimulus presentation). Looking could vary from brief scans to extended visual contact for several seconds. Each look at the model was counted as one occurrence of LOOK. (ii) LPS. The mouth is opened and closed quickly. The mouth is not opened to its full extent (but generally to one-third). LPS may be combined with TP. Each opening of the mouth was counted as one occurrence of LPS. Occurrences of TP were scored separately. (iii) MO. The mouth is opened for at least half of its total opening span, and usually only once. MO is performed more slowly than LPS, and the mouth is maintained open for a slightly longer period. Each opening of the mouth was counted as one occurrence of MO. TPs could occur in combination with MO. Occurrences of TP were scored separately. (iv) TP. Forward movements of the tongue so that it crosses the inner edge of the lower lip. Each thrust was scored as one occurrence of TP. (v) HO. Opening and closing of a hand without arm movements. Generally, fingers are tightened around support (usually fleece fabric) with a whole hand grip. Each opening and closing of one hand was scored as one occurrence of HO. (vi) Move arm and grasp (MOVE-HO). Grip is released from support, arm moves toward another area on support, and the support is gripped again. Each of these sequences was counted as one occurrence of MOVE-HO.

### Statistical analysis: Attention toward the biological and nonbiological objects.

Wilcoxon paired tests were used to compare the amount of attention (LOOK) that the infant paid to the biological (face or hand) or to the nonbiological stimulus (disk) during the baseline and the stimulus periods. For each animal, scores obtained in the three facial conditions (LPS, TP, and MO) were averaged.

### Statistical analysis: Stimulus versus baseline period.

In each condition, we assessed whether the monkeys' behavior that matched the behavior provided by the experimenter (target behavior) was performed by the infant with higher frequency during stimulus periods than during baseline. For this purpose, we compared the frequency of each behavior displayed during the stimulus period with that displayed during baseline. The frequency of each behavior in the stimulus and baseline periods were compared with Wilcoxon paired tests.

### Comparison of the monkeys' behavior during each experimental condition with the biological control (EYE) condition.

To compare the frequency of the matched behavior in a condition with that scored in the control, we calculated for each infant the difference in frequency between the matched behavior displayed during the stimulus period and the baseline period. A negative score indicated that a behavior was observed more frequently during baseline; a positive score indicated that a behavior was observed more frequently during the stimulus period. Wilcoxon tests were used to compare the score for each matched behavior displayed in a specific condition with the score of the same behavior displayed in the control condition (EYE condition). We ran the same comparison between each stimulus period and the same behavior displayed in the nonbiological control (DISK condition). To exclude that the frequency of the matched behavior could increase as a consequence of neonate general arousal for seeing a specific mouth or hand movement, we compared the score of each matched behavior displayed in a specific condition with that obtained in the other conditions (Wilcoxon paired tests).

## Supporting Information

Video S1 3-d-Old Macaque Infant Imitating Mouth Opening This video illustrates a 3-d-old infant male macaque responding to the experimenter mouth gesture.
(3.3 MB AVI)

Video S2 3-d-Old Macaque Infant Imitating Tongue Protrusion This video illustrates a 3-d-old infant female macaque responding to the experimenter's TP.
(4.0 MB AVI)

Video S3 Lip Smacking Exchanges in a Naturalistic Setting between Mother and Infant Macaques This video was taken at the field station in Poolesville, Maryland, United States (National Institute of Child Health and Human Development). It depicts a face-to-face mother–infant LPS exchange with the mother initiating the interaction. The infant is less than 10 d old.
(2.5 MB AVI)

## Abbreviations

EYE: eyes opening
HO: hand opening
LPS: lip smacking
MO: mouth opening
TP: tongue protrusion
